# Phase-Separation-Engineered Porous Polyimide Fibers via Wet Spinning for Superior Thermal Insulation

**DOI:** 10.3390/molecules31183231

**Published:** 2026-09-13

**Authors:** Ruihong Sun, Fujuan Liu

**Affiliations:** National Engineering Laboratory for Modern Silk, College of Textile and Clothing Engineering, Soochow University, 199 Ren-Ai Road, Suzhou 215123, China; 20245215009@stu.suda.edu.cn

**Keywords:** PI fiber, wet spinning, phase separation, porous structure, thermal insulation

## Abstract

Personal thermal management (PTM) textiles can reduce building energy consumption and improve personal comfort, yet their practical application is constrained by the inherent trade-off between flexibility, thermal insulation, and mechanical strength. Herein, porous single-component polyimide (PI) fibers were fabricated via coagulation bath-modulated wet spinning of 3,3′,4,4′-benzophenone tetracarboxylic dianhydride (BTDA)–4,4′-oxydianiline (ODA) poly(amic acid) (PAA). By tuning the EtOH/H_2_O ratio (20/80–60/40) and winding speed (2.6–13.1 mm/s), the fiber cross-sectional morphology evolves from finger-like macropores to uniform spongy networks, with diameters controllable from 120 to 335 μm. The PI porous fibers exhibit a maximum tensile strength of 56.79 MPa, elongation at break of 13.89%, toughness of 4.98 MJ/m^3^, and thermal conductivity as low as 0.043 W·m^−1^·K^−1^. The highly imidized structure was confirmed by FTIR (imidization index = 0.848), and TGA revealed high thermal stability with 5% weight loss temperatures of 491 °C (N_2_) and 488 °C (air). Compared with commercial insulators, a single-layer PI fabric (0.892 mm) shows thermal insulation comparable to that of the thicker aramid 1313 fabric (1.588 mm) under the same 100–200 °C hot-plate conditions, while also exhibiting self-extinguishing behavior equivalent to that of aramid 1313. The 5-layer PI stack (3.637 mm) is only half as thick as glass fiber cotton (7.342 mm) but retains 84–91% of its temperature difference, delivering 1.7–1.8 times higher thickness-normalized insulation efficiency. The ultrathin porous PI fabrics integrate robust mechanical performance, excellent thermal shielding, and flame retardancy, and are promising for extreme-environment thermal management including fire protection, spacecraft thermal control, and battery insulation.

## 1. Introduction

High-performance thermal insulation fibers are essential core materials for extreme-environment thermal protection, playing a key role in advanced fields such as aerospace equipment, special protective equipment and new energy battery safety protection. As a typical high-performance polymer material, polyimide (PI) fibers possess excellent thermal stability, mechanical strength, and radiation resistance, rendering them ideal candidates for thermal protection in extreme environments [1,2,3,4], such as aerospace vehicles [5,6], firefighting suits [7], and battery thermal runaway protection systems [8]. In particular, PI fibers exhibit inherent flame retardancy with a limiting oxygen index (LOI) typically exceeding 38%, which is substantially higher than that of conventional flame-retardant fibers like Nomex [9,10]. Mir et al. [1] systematically reviewed the design principles of high-temperature PI aerogels, emphasizing strategies to overcome challenges including thermal shrinkage, structural collapse, and material brittleness. Recent comprehensive reviews have further summarized the heat transfer mechanisms, fabrication routes, and performance enhancement strategies of porous PI thermal insulation materials, covering chemical structural design, process optimization, and composite construction [8,11]. However, conventional dense PI fibers exhibit relatively high thermal conductivity, limiting their thermal insulation performance. In contrast, PI materials with high porosity (porosity > 90%) suffer from severe degradation in mechanical properties, with tensile strength of only 0.15–30 MPa [12,13,14]. Common methods for creating porous fibers, such as electrospinning [15,16], template-assisted synthesis [17], and thermally induced phase separation [18,19], are constrained by low productivity, complex processes, high energy consumption, and poor continuous production capability [20,21]. Moreover, the dehydration reaction occurring during the thermal imidization of two-step PI precursor fibers tends to generate micropores and structural defects, further deteriorating the mechanical performance. To address the inherent trade-off between thermal insulation capacity and mechanical robustness, recent studies have focused on wet spinning combined with coagulation bath regulation, which offers a versatile approach for tailoring the pore structure of PI fibers [13,22]. For example, core–shell-structured PI aerogel fibers with a “porous cortex dense core” configuration [23,24,25] were prepared by coaxial wet spinning based on hierarchical phase separation, achieving a high tensile strength of 55.2 MPa and a low thermal conductivity of 37.2 mW/(m·K) [12]. Additionally, the addition of reinforcing phases such as polyacrylonitrile (PAN) [9,26] and polyacrylonitrile fibers (PANFs) [27] can effectively enhance the structural stability and strength of porous fibers.

The key to porous fiber fabrication via wet spinning lies in the regulation of phase separation behavior through adjusting coagulation bath composition, which in turn determines the microporous structure of the resultant fibers [13,22,28,29]. The ratio of non-solvents (e.g., water/ethanol) in the coagulation bath significantly affects the cross-sectional morphology of the fibers [30]. Specifically, a high non-solvent ratio tends to produce finger-like macrovoids, while an appropriate ratio facilitates the generation of a uniform sponge-like network. For instance, Dong et al. [31] found that the ethanol/water ratio can effectively regulate the internal void structure of as-spun PI fibers. They further constructed a ternary phase diagram of the water/N-methyl-2-pyrrolidone (NMP)/PI system based on Flory–Huggins theory, revealing the regulatory mechanism of coagulation temperature on the final fiber structure by modulating the phase separation pathway. Moreover, the mechanical properties of wet-spun fibers are governed by the synergistic effect of coagulation bath composition and winding speed. Elokhovskii et al. [32] reported that an ethylene glycol/ethanol mixture (50/50 vol%) serves as the optimal coagulation bath system for producing defect-free PI fibers. Although the wet spinning of BTDA-ODA PI fibers was demonstrated in early work by Dorogy and St. Clair [33], there remains a lack of systematic investigation on the pore structure transition behavior regulated by coagulation bath composition, as well as the coupled effects of such structural evolution on the mechanical and thermal insulation properties of single-component BTDA-ODA PI fibers [34].

In this work, PI fibers were prepared via wet spinning using BTDA-ODA polyamic acid (PAA) as the precursor. Unlike previous studies that focused on other PI systems, this work systematically investigates the effects of the EtOH/H_2_O coagulation bath ratio (ranging from 20/80 to 60/40) and winding speed (2.6–13.1 mm/s) on the structural transition from finger-like macrovoids to sponge-like networks in single-component BTDA-ODA PI fibers, as well as the corresponding synergistic variation in mechanical and thermal insulation properties. The relationship between the phase separation mechanism induced by the coagulation bath and the resulting fiber performances is comprehensively discussed. Although this study focuses on single-component PI fibers, the obtained findings provide a reliable experimental basis for the subsequent development of high-performance core–shell-structured PI fibers.

## 2. Results and Discussion

### 2.1. Winding Speeds

**Figure 1:**
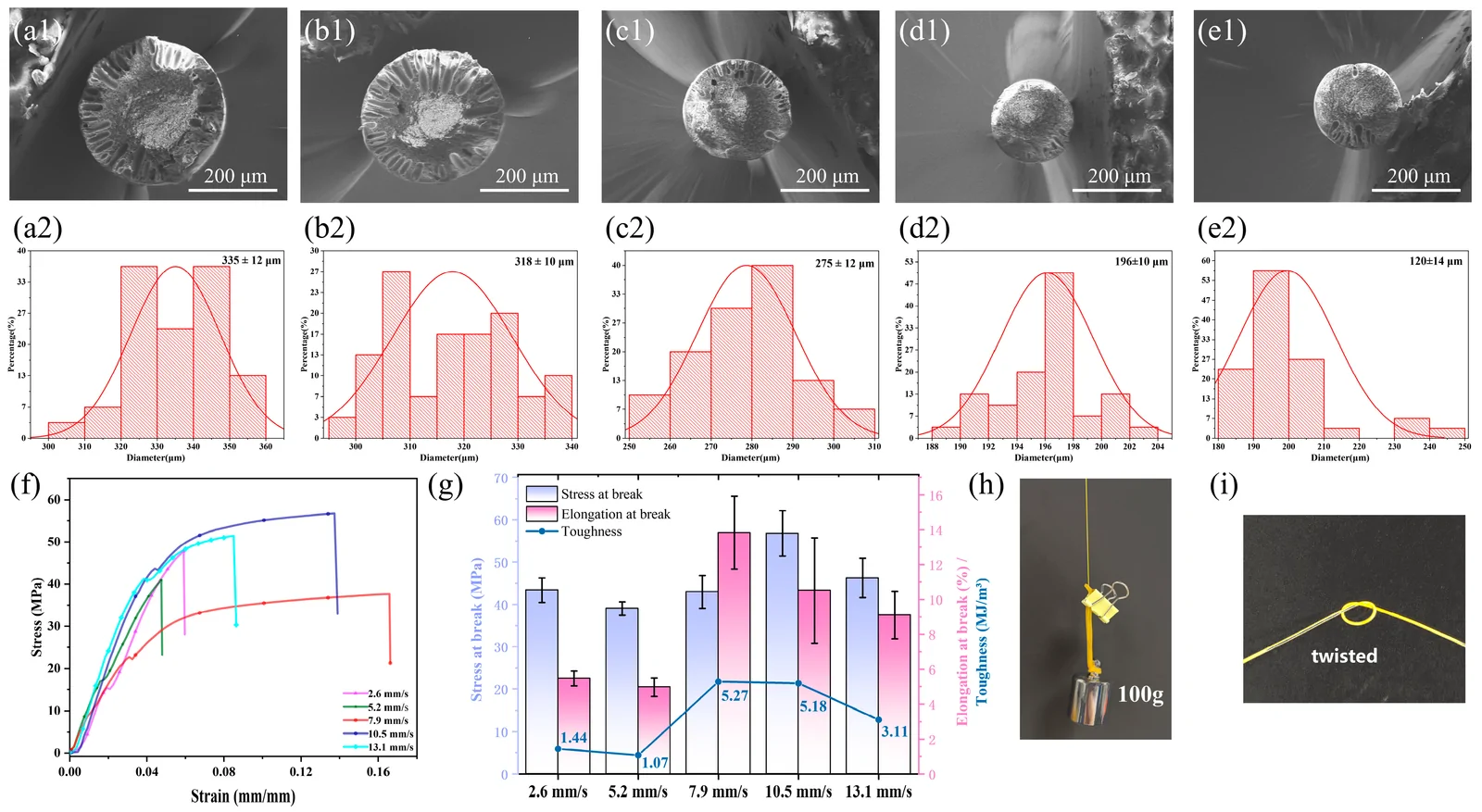
Cross-sectional SEM images (**a1**–**e1**) and corresponding diameter distribution histograms (**a2**–**e2**) of PI fibers spun at winding speeds of 2.6, 5.2, 7.9, 10.5 and 13.1 mm/s, respectively; (**f**) stress–strain curves; (**g**) stress at break, elongation at break, and toughness; (**h**) fiber bearing a 100 g load; (**i**) twisted fiber.

Figure 1 presents the microstructure and mechanical performance of PI fibers fabricated at a fixed EtOH/H_2_O ratio of 40/60 and a constant extrusion velocity of 8 mL·h^−1^. The cross-sectional SEM images of the PI fibers obtained at different winding speeds are shown in Figure 1(a1–e1). Correspondingly, Figure 1(a2–e2) show that the fiber diameter decreases monotonically from 335 μm to 120 μm with the increase in winding speed, which originates from the enhanced axial tension that causes radial fibers during spinning. The typical stress–strain curves of as-prepared fibers having observable plastic deformation are exhibited in Figure 1f. The breaking stress declines overall from 43.37 MPa at 2.6 mm/s to 42.97 MPa at 7.9 mm/s (despite a minor rebound at 5.2 mm/s), then rises sharply to a maximum value of 56.79 MPa at 10.5 mm/s, and finally decreases to 46.26 MPa at 13.1 mm/s (Figure 1g). The initial reduction at low speeds arose from insufficient molecular orientation and defective coarse pore structures of nascent fibers, which promoted stress concentration. By contrast, the subsequent strength improvement resulted from the effective molecular chain alignment and increased crystallinity, which enhanced the axial load-bearing capacity of the fibers. The elongation at break remains at a relatively low level of 4.96–5.76% within the speed range of 2.6–5.2 mm/s, reaches a peak value of 13.63% at 7.9 mm/s, and then decreases to 10.51% and 9.10% at 10.5 and 13.1 mm/s, respectively (Figure 1g). The peak at 7.9 mm/s corresponds to the optimal drawing ratio, where refined nanopore walls can effectively dissipate external energy through plastic deformation. The subsequent decline resulted from excessive molecular orientation and reduced free volume, which increased the brittleness of the fiber microstructure. Toughness, defined as the integrated area under the stress–strain curve, exhibits a similar trend and reaches its maximum value of 5.28 MJ/m^3^ at 7.9 mm/s (Figure 1g). Figure 1h,i demonstrate that the PI fiber can stably suspend a 100 g weight and withstand twisting deformation, confirming its excellent flexibility and mechanical load-bearing performance.

To assess the practical potential of the porous PI fibers for thermal protective textiles, their mechanical properties were compared with those of commercial polyester and aramid fibers (Table 1). Although the tensile strength of the PI fibers is inferior to that of dense commercial fibers, this is an inherent consequence of their porous architecture. Notably, the PI fibers exhibit elongation (~14%) comparable to PET and substantially higher ductility than Kevlar (~3.5%).

The above results indicate that winding speed significantly affects the mechanical properties of the PI fibers. Samples spun at 7.9 mm/s achieve a desirable balance between tensile strength and elongation at break, accompanied by the maximum toughness (5.28 MJ/m^3^). Accordingly, this condition was selected for all subsequent experiments.

### 2.2. Optimization of Coagulation Bath Conditions

#### 2.2.1. Morphology of PI Fibers

**Figure 2:**
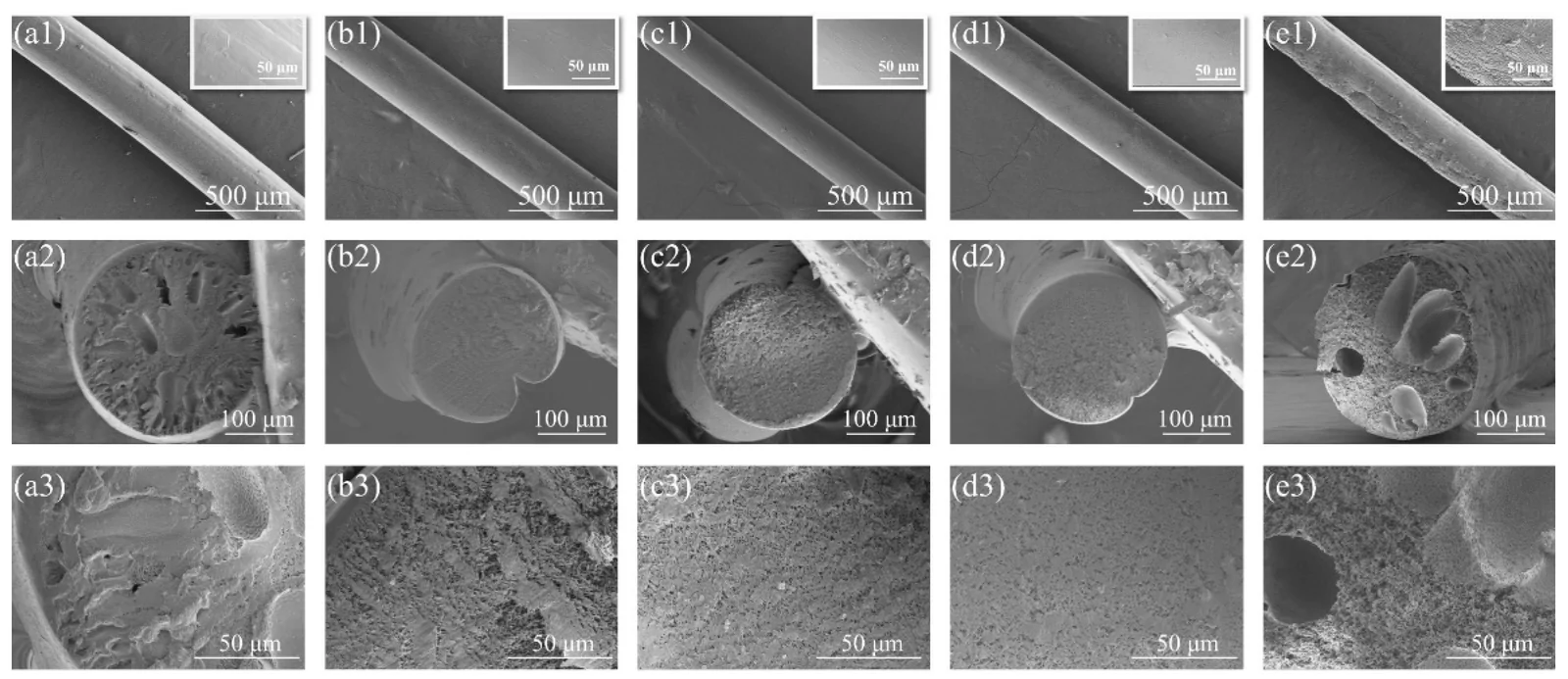
Morphology of PI fibers (V_e_ = 8 mL/h, V_w_ = 7.9 mm/s). (**a1**–**e1**), (**a2**–**e2**) and (**a3**–**e3**) SEM images of the fiber surfaces, cross-sections and corresponding magnified views at different coagulation bath compositions (EtOH/H_2_O = 20/80, 30/70, 40/60, 50/50 and 60/40, respectively).

The composition of the coagulation bath imposes a remarkable effect on the fiber morphology, as visualized by the SEM images (Figure 2). At a high water content (EtOH/H_2_O = 20/80), water acts as a strong non-solvent, inducing instantaneous phase separation. The outer skin layer rapidly solidifies into a dense structure. However, the excessively fast diffusion rate gives rise to microcracks on the fiber skin, and elliptical micropores alongside large voids emerge across the cross-section. When the water ratio is adjusted to an EtOH/H_2_O proportion of 30/70–50/50, the fibers display smooth surfaces together with a sponge-like network cross-section, which indicates a moderate phase separation rate and uniform structure within this range. As the ethanol fraction further increases to 60/40, ethanol serves as a weak non-solvent and slows down phase separation. The skin layer develops slowly with poor mechanical integrity, while the fiber core stays in a swollen state for a prolonged period, resulting in a loose internal architecture. During the subsequent drying process, shrinkage occurs, and the fragile skin cannot resist the contraction stress from the core, triggering buckling instability. Consequently, axial micro-wrinkles are generated on the fiber surface.

**Figure 3:**
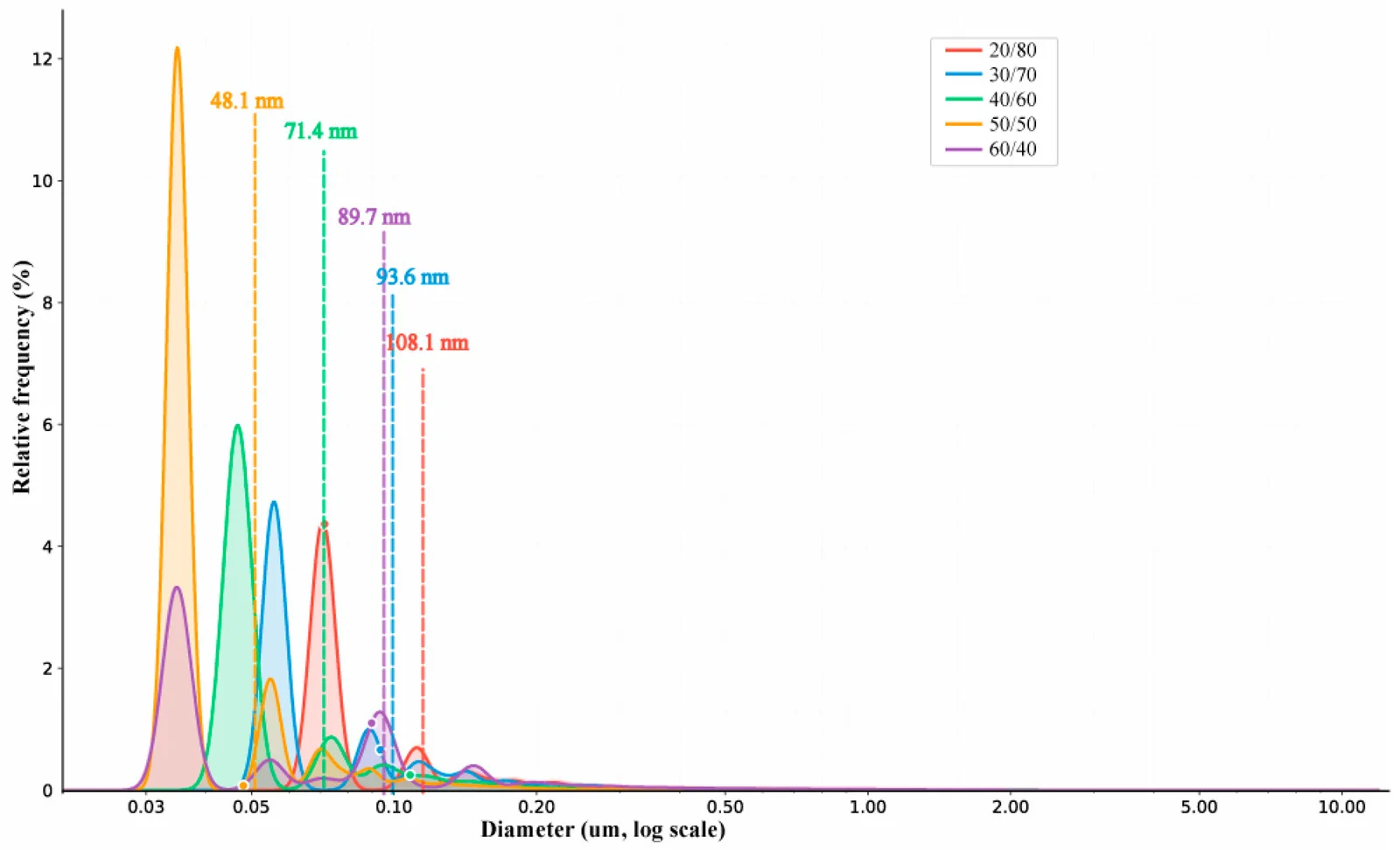
Pore size distribution curves of samples with different ratios. The dashed lines denote the mean pore diameters.

To quantitatively characterize the pore architecture of fibers, ImageJ (version 1.53e, NIH, Bethesda, MD, USA) analysis was performed on the SEM cross-sectional images. Figure 3 presents the pore size distribution curves, and Table 2 summarizes the key structural parameters. These parameters represent 2D cross-sectional area fractions and are used for comparing the morphology of the samples. The pore size distributions vary systematically with the composition of the coagulation bath. From the 20/80 to 40/60 group, the peak shifts toward smaller pore sizes from approximately 70.8 nm to 46.8 nm with increasing intensity, indicating progressive pore refinement. The 50/50 sample shows the sharpest single peak (12.18%), corresponding to the finest pore morphology, while the 60/40 sample displays a bimodal distribution with a distinct secondary peak at around 93 nm, indicative of a heterogeneous internal structure. The lines in Figure 3 denote the weighted mean pore diameters, which reflect the right-skewed feature of the pore size distributions. Further quantitative statistics (Table 2) reveal that the 20/80 sample possesses the highest open area fraction (34.71%) on the cross-sectional plane and largest mean pore diameter (108.1 nm), whereas the 50/50 sample shows the smallest mean pore diameter (48.1 nm) and lowest open area fraction (21.14%). These structural features, especially open area fraction and mean pore size, will be correlated with the thermal conductivity in the following discussion.

#### 2.2.2. Mechanical Properties

**Figure 4:**
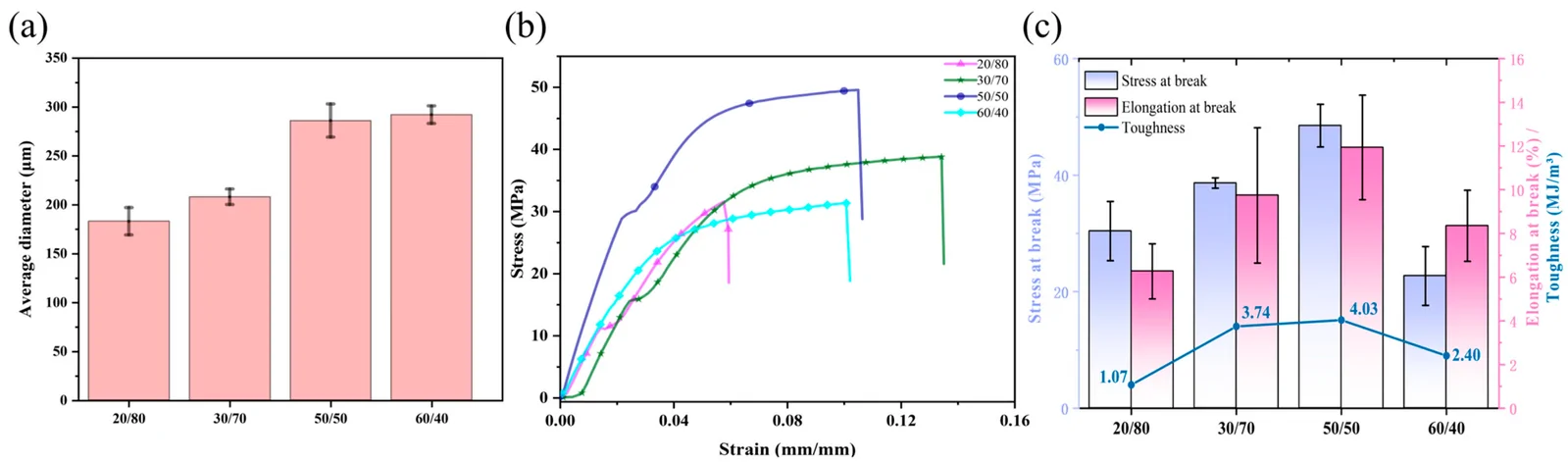
Diameter and mechanical properties of PI fibers. (**a**) Average diameter; (**b**) stress–strain curves; (**c**) stress at break, elongation at break, and toughness.

Figure 4 illustrates the effect of the EtOH/H_2_O ratio on fiber diameter and mechanical properties. As the ethanol content increases from 20% to 60%, the average diameter monotonically increases from 183 μm to 292 μm (Figure 4a). This may be attributed to the weaker coagulation power of ethanol, which slows solvent–nonsolvent exchange, reduces radial contraction, and keeps the fiber swollen for a longer time. The breaking stress first increases from 30.40 MPa (EtOH/H_2_O = 20/80) to a maximum of 48.50 MPa at 50/50, then dropped sharply to 30.27 MPa at 60/40 (Figure 4b). The initial rise results from a shift from instantaneous to moderate phase separation, which reduces structural defects (e.g., microcracks and large voids) and facilitates the formation of a uniform sponge-like network. The subsequent decline at 60/40 can be ascribed to delayed phase separation, producing fragile wrinkled skin layers and a loose fiber core. Both features act as stress concentrators. Elongation at break exhibits a similar trend, reaching a peak value of 13.89% at 40/60 (Figure 1g), and then decreasing to 11.93% and 8.34% at 50/50 and 60/40, respectively. The maximum elongation at 40/60 reflects an optimal pore structure, wherein the sponge-like network with refined pore walls dissipates energy through plastic deformation. A higher ethanol content (60/40) leads to excessive loosening and skin buckling, thereby reducing ductility.

Toughness was calculated by integrating the area under each stress–strain curve. The 40/60 sample shows the highest toughness of 4.98 MJ/m^3^, which originates from its well-balanced tensile strength (42.20 MPa) and elongation at break (13.89%) (Figure 1g). Although the 50/50 sample has the highest strength (48.50 MPa), its reduced elongation (11.93%) results in a slightly lower toughness of 4.03 MJ/m^3^ (Figure 4c). This confirms that optimal toughness is achieved through a synergistic balance between strength and ductility, rather than by maximizing a single mechanical parameter alone.

Based on the quantitative toughness analysis, the optimal coagulation bath composition for balancing strength and toughness lies within the 40/60–50/50 range, and the maximum toughness of 4.98 MJ/m^3^ is attained under the 40/60 condition.

#### 2.2.3. Thermal Imaging

**Figure 5:**
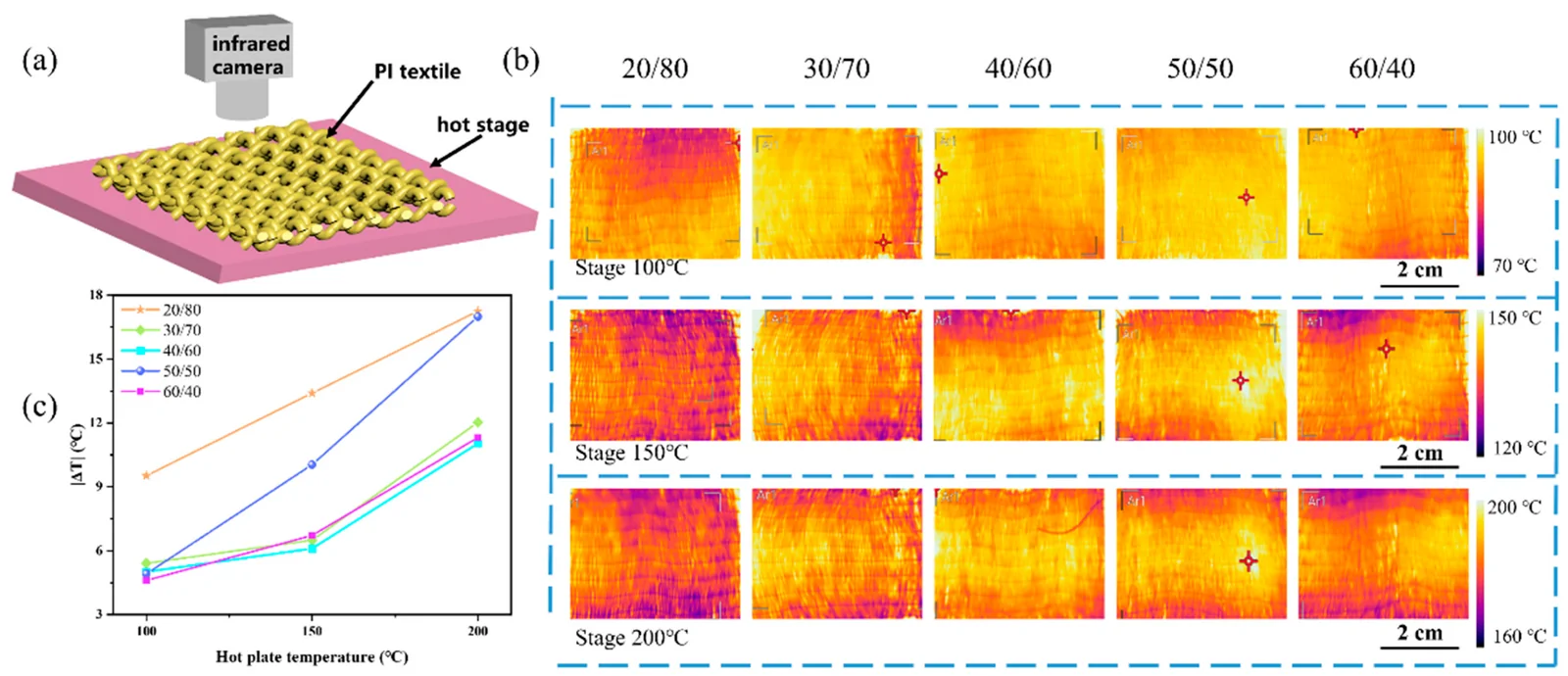
(**a**) Schematic illustration of the thermal insulation test setup; (**b**) infrared thermal images of PI fabrics at 100 °C, 150 °C and 200 °C (the "Ar1" labels and cross markers are software-generated indicators); (**c**) temperature differences as a function of hot-plate temperature (R^2^ > 0.95 for all samples).

Woven fabric samples (5 cm × 5 cm) were placed on a hot plate and heated at 100, 150, and 200 °C for 120 s, followed by surface temperature measurement. Table 3 summarizes the surface temperatures of the PI fiber fabrics after heating, and Figure 5 shows the corresponding infrared thermal images. The fiber prepared under the EtOH/H_2_O ratio of 20/80 achieved the maximum temperature difference ΔT (hot-plate temperature minus surface temperature) at all three test temperatures, reaching 9.52, 13.4 and 17.25 °C, respectively, which exceeded those of other samples. The 50/50 fiber gave intermediate ΔT values of 10.04 and 16.99 °C at 150 and 200 °C, while the other groups (30/70, 40/60 and 60/40) showed considerably lower differences.

Based on the steady-state heat conduction described by Fourier’s law (q = λ_eff_ ΔT/d), the measured temperature difference ΔT directly reflects the variation in the effective thermal conductivity λ_eff_ under the assumption of a constant heat flux. Taking the 20/80 sample as an example, ΔT increases nearly linearly with hot-plate temperature (R^2^ = 0.9996), suggesting that λ_eff_ does not vary significantly across the tested temperature range. This apparent stability can be partly attributed to the porous structure, where trapped air suppresses gas convection while the solid skeleton limits thermal pathways. It should be noted, however, that the linear ΔT dependence alone does not strictly prove a temperature-independent λ_eff_, since heat flux, contact resistance, and radiative losses may also evolve with temperature. The slight deviation from linearity at elevated temperatures suggests a moderate enhancement of radiative heat transfer within the pores, which is a typical characteristic of porous thermal insulation materials.

The distinct thermal insulation performance across the samples originates from the morphological differences induced by the coagulation bath composition. The 20/80 sample, featuring a dense skin layer and macroporous core, achieves the maximum ΔT and thus the optimal insulation performance. In comparison, the sponge-like networks of the 30/70 and 40/60 samples, as well as the structural defects present in the 60/40 sample, progressively deteriorate the insulation. Quantitative analysis of these structure–property relationships is provided in Section 2.2.4 and Section 2.2.5.

#### 2.2.4. Thermal Conductivity

To quantitatively evaluate the thermal insulation performance, the thermal conductivity of the porous PI fibers was experimentally measured. The resultant thermal conductivity values ranging from 0.043 to 0.053 W·m^−1^·K^−1^ are listed in Table 4. The fiber fabricated at an EtOH/H_2_O ratio of 20/80 exhibits the lowest thermal conductivity (0.043 W·m^−1^·K^−1^) and the highest thermal resistance (0.020 m^2^·K·W^−1^), which accounts for its best insulation performance. In contrast, the 40/60, 50/50, and 60/40 samples show relatively higher thermal conductivities of 0.050–0.053 W·m^−1^·K^−1^. The variation trend of thermal conductivity (20/80 < 30/70 < 40/60 < 60/40 < 50/50) is highly consistent with the ΔT profile acquired via infrared thermography (Figure 5b,c). These two independent measurement results mutually corroborate each other, demonstrating that the intrinsic pore architecture governs thermal insulation performance of the fibers.

The bulk porosity measured by the n-butanol method ranges from 45% to 77% (Table 4). However, no simple linear negative correlation exists between bulk porosity and thermal conductivity. The 20/80 sample achieves the lowest λ value (0.043 W·m^−1^·K^−1^) with a moderate bulk porosity (59.55%), while the 60/40 sample possesses a higher bulk porosity (~70%) but a higher λ (0.052 W·m^−1^·K^−1^). This apparent contradiction can be explained by the quantitative pore structure parameters (Table 2). The 20/80 sample exhibits the largest mean pore diameter (108.1 nm) and the highest open area fraction (34.71%), which favors air-dominated heat conduction and suppresses solid-phase heat transfer. In contrast, despite its high bulk porosity, the 60/40 sample shows a lower open area fraction (23.27%) and a bimodal pore distribution. The structural defects in its surface layer introduce thermal bridges that compromise the insulation benefit. The 50/50 sample, with the smallest mean pore diameter (48.1 nm) and the lowest open area fraction (21.14%), exhibits the highest thermal conductivity (0.053 W·m^−1^·K^−1^), indicating that excessive pore refinement reduces air-filled voids and builds continuous solid conduction pathways, thereby deteriorating thermal insulation performance. Combined analysis of bulk porosity (Table 4) and morphological parameters (Table 2) confirms that the thermal insulation behavior is governed by the synergistic effects of pore size, open area fraction, and structural uniformity, rather than bulk porosity individually.

#### 2.2.5. Heat Transfer Mechanism Analysis

To further evaluate the heat-transfer contributions and assess the structural interpretation, theoretical bounding models were adopted. For the tested temperature range (≤200 °C) and pore size scale (48–108 nm), natural convection is fully suppressed owing to a considerably low Rayleigh number (Ra ≪ Ra_c_), while thermal radiation is negligible, with a radiative thermal conductivity lower than 10^−5^ W·m^−1^·K^−1^. The thermal conduction of internal gas is approximated to that of static air (λ_air_ = 0.026 W·m^−1^·K^−1^), and the Knudsen number (Kn ≈ 0.6–1.4) confirms that gas heat transfer falls within the transition regime. It is acknowledged that, at Kn ≈ 0.6–1.4, gas-wall interactions may reduce the effective gas thermal conductivity below the bulk air value. A rigorous Knudsen correction would require detailed pore size distribution data and accommodation coefficients, which are beyond the scope of this work. Nevertheless, because all samples share a similar Kn range and a narrow pore size window (48–108 nm), any systematic reduction in λ_air_ would affect all samples to a comparable extent. Consequently, the use of a constant λ_air_ as a consistent baseline preserves the relative ranking of the back-calculated solid-phase contributions and remains acceptable for this semi-quantitative bounding analysis. Accordingly, the experimentally measured thermal conductivity is predominantly determined by gas and solid conduction, among which solid conduction is highly dependent on the microstructural characteristics of the porous PI fibers.

The parallel model (upper bound, continuous solid phase) and the series model (lower bound, interrupted solid phase) were adopted to bracket the admissible range of effective thermal conductivity:

(1) $$\lambda_{\mathrm{parallel}}=\varphi \cdot \lambda_{\mathrm{air}\;}+(1-\varphi )\cdot \lambda_{\mathrm{solid}}$$

(2) $$\lambda_{\mathrm{series}}={\;(\frac{\varphi }{\lambda_{\mathrm{air}}}+\frac{1-\varphi }{\lambda_{\mathrm{solid}}})}^{-1}$$

where φ is the bulk porosity from Table 4 (n-butanol method), λ_air_ = 0.026 W·m^−1^·K^−1^, and λ_solid_ = 0.25 W·m^−1^·K^−1^. The effective solid thermal conductivity can be back-calculated as:

(3) $$\lambda_{\mathrm{eff}\;}=\;\frac{\lambda_{\mathrm{measured}}-\varphi \cdot \lambda_{air}}{1-\varphi }$$

where λ_eff_ is an equivalent solid-phase conductivity defined by Equation (3); it is not the measured total conductivity and is not bounded by λ_series_.

While the back-calculated λ_eff_ values offer semi-quantitative support for the suppression of solid conduction, the mechanistic interpretations, such as interruption of solid conduction pathways and thermal bridging, are inferred from the corresponding morphological features observed by SEM. In this discussion, bulk porosity (φ), thermal conductivity (λ_measured_), and pore size distribution are experimentally measured quantities, whereas pore connectivity, skeleton continuity, and thermal bridging are morphological interpretations based on SEM observations. Accordingly, Table 5 summarizes the calculated model results. The 20/80 sample shows the lowest measured thermal conductivity (0.043 W·m^−1^·K^−1^); its back-calculated λ_eff_ (0.068 W·m^−1^·K^−1^, 27% of λ_solid_) is consistent with interrupted solid conduction pathways. This low λ_eff_ is consistent with the morphological observation that the macroporous structure may effectively interrupt solid conduction pathways. In contrast, the 60/40 sample gives the highest back-calculated λ_eff_ (0.114 W·m^−1^·K^−1^, 46% of λ_solid_), which is plausibly associated with the interconnected solid skeleton visible in the SEM images, potentially forming thermal bridges that facilitate solid conduction. Within this bounding framework, these findings, combined with the pore structure parameters presented in Section 2.2.1, suggest a consistent structure-performance trend for the porous PI fibers: the thermal conductivity is not governed by total porosity alone, but is also influenced by pore size distribution, open area fraction, and structural uniformity. It should be noted, however, that the parallel and series models serve as upper and lower bounds rather than as an independent quantitative separation of the solid- and gas-phase contributions. The back-calculated λ_eff_ values are useful for comparative purposes across samples but should not be regarded as an absolute determination of the solid-phase thermal conductivity.

#### 2.2.6. Thermal Insulation

**Figure 6:**
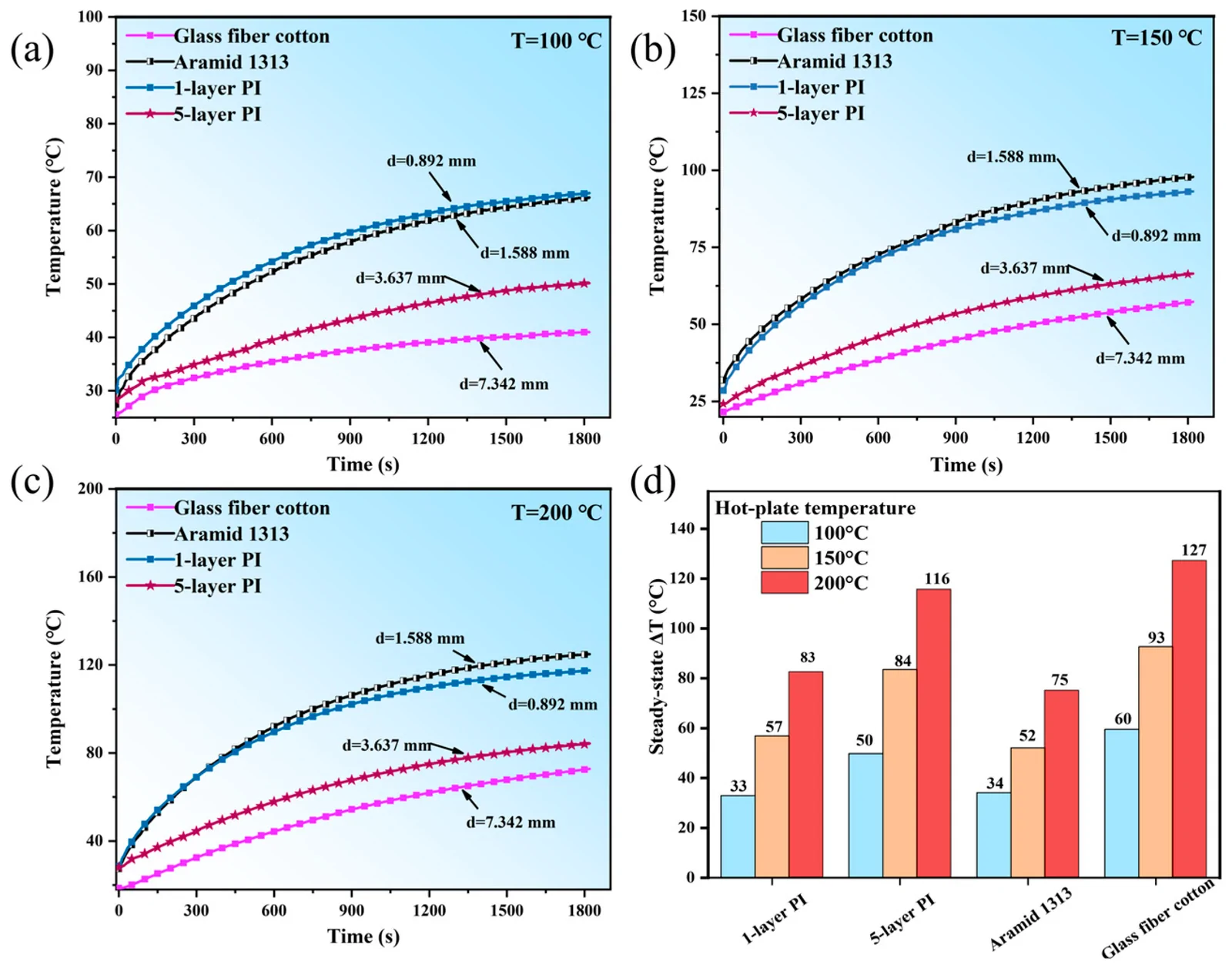
Thermocouple tests and flame-retardant performance of PI fabrics. (**a**–**c**) Time-dependent temperature profiles measured via thermocouples at 100 °C, 150 °C and 200 °C, respectively; (**d**) steady-state temperature difference (ΔT =T_hot-plate_ − T_max_).

The time-dependent surface temperatures of four fabric samples measured at hot-plate temperatures of 100, 150, and 200 °C are shown in Figure 6a–c. At 100 °C, the 1-layer PI fabric reaches a surface temperature of 67.1 °C, comparable to that of aramid 1313 (65.8 °C), despite being only 56% of the thickness of its aramid counterpart. At 150 °C and 200 °C, the surface temperatures of PI fabric reach 93.1 and 117.5 °C, respectively, which are lower than those of aramid 1313 (97.9 and 124.9 °C) under the same test conditions, suggesting a better thermal blocking performance at elevated temperatures when compared on an equal layer number. For the multi-layer configuration, the 5-layer PI stack (3.637 mm, ~50% of the thickness of glass fiber cotton) retains 84–91% of the temperature difference achieved by glass fiber cotton, although its absolute insulation is slightly lower due to the much smaller thickness. Notably, when normalized by thickness, the PI stack exhibits a thermal insulation efficiency approximately 1.7–1.8 times that of glass fiber cotton (Figure 6d). These results demonstrate that the porous PI fabric can provide thermal protection comparable to that of aramid fabric under the specific hot-plate conditions employed, and offers a distinct advantage in thickness-specific efficiency over glass fiber cotton.

Based on a comprehensive evaluation of mechanical and thermal insulation performance, the 40/60 ratio offers the best overall performance. Its thermal conductivity is merely 0.007 W·m^−1^·K^−1^ higher than that of the 20/80 sample, indicating comparable thermal insulation, while its toughness (4.98 MJ/m^3^) and elongation at break (13.89%) are substantially superior to those of the 20/80 sample. Compared with the 50/50 sample, the 40/60 sample exhibits lower thermal conductivity and higher toughness, along with a clear advantage in elongation at break. Therefore, the 40/60 ratio is identified as the most promising coagulation bath condition.

### 2.3. Chemical Structure and Thermal Stability

**Figure 7:**
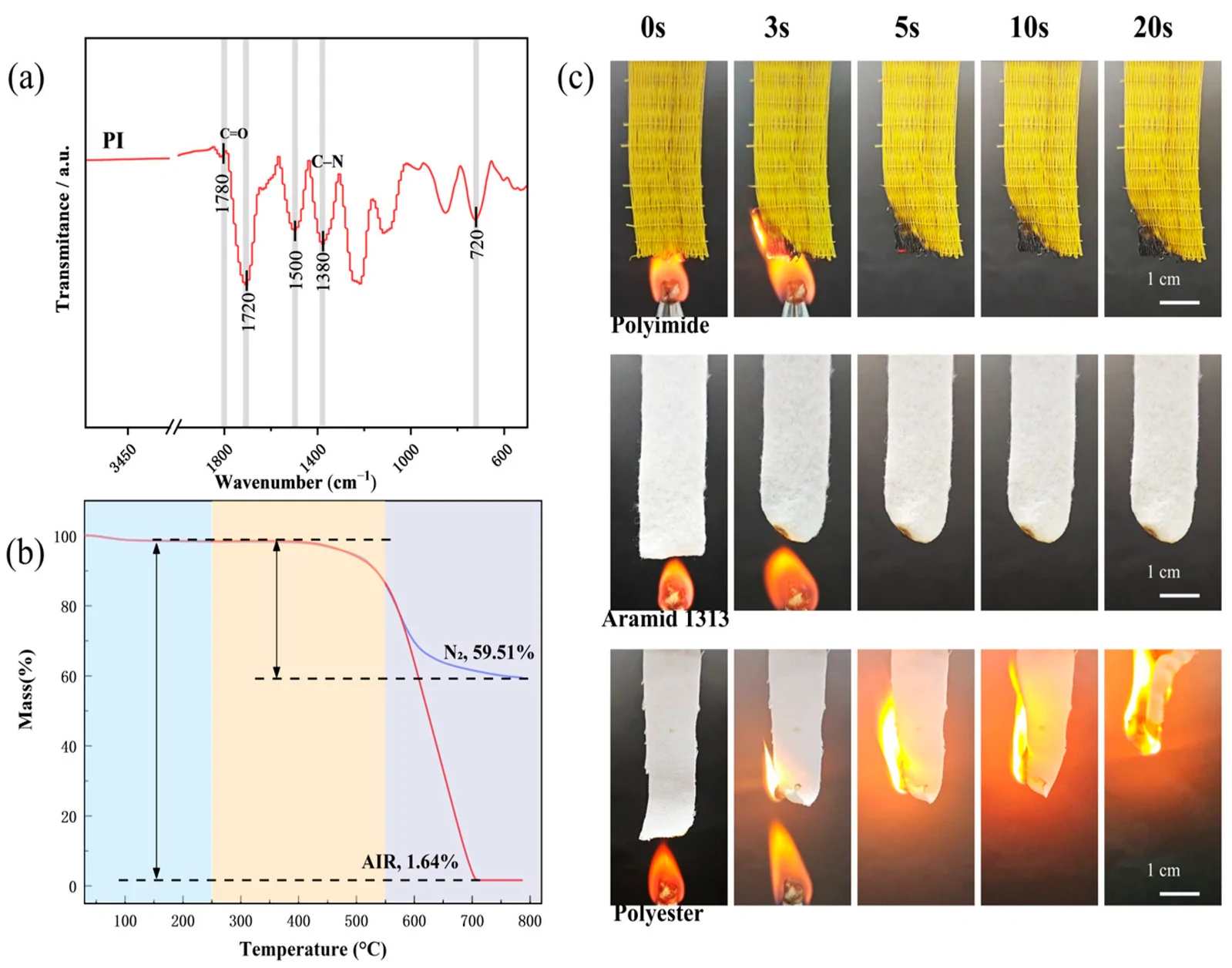
(**a**) FTIR spectra of PI fibers; (**b**) TGA curves under nitrogen and air atmospheres (the dashed lines and arrows indicate the mass loss platforms and final residual mass of 59.51% in N_2_ and 1.64% in Air); (**c**) vertical flame tests for PI and reference fabrics.

The chemical structure of the synthesized PI fibers was confirmed by FTIR spectroscopy. As shown in Figure 7a, the characteristic absorption bands of the imide ring appear at ~1780 cm^−1^ (asymmetric C=O stretching), ~1720 cm^−1^ (symmetric C=O stretching), ~1380 cm^−1^ (C–N stretching), and ~725 cm^−1^ (imide ring bending), consistent with the typical FTIR fingerprints of highly imidized BTDA-ODA polyimide reported in the literature [38]. To quantitatively evaluate the imidization degree, the imidization index (II) was calculated as the ratio of S_1380_ to S_1500_, yielding a value of 0.848 (S_1380_ = 0.268, S_1500_ = 0.316). Using a fully imidized reference sample, the relative imidization degree (ID) was determined to be 94.2%, confirming the near-complete imidization of the PI, which is essential for achieving the high thermal stability and mechanical integrity of the resulting PI fibers.

The thermal stability of the PI fibers was evaluated by TGA under both nitrogen and air atmospheres (Figure 7b). The TGA curves show negligible weight loss (<0.02%) in the 100–150 °C range, confirming that the fibers were thoroughly dried and free from residual solvent or adsorbed moisture that could otherwise compromise the thermal conductivity measurements. The characteristic thermal degradation parameters are summarized in Table 6. In nitrogen, the 5% and 10% weight loss temperatures (T_d,5%_ and T_d,10%_) are 491.47 °C and 534.02 °C, respectively, with a high char residue of ~59.5% at 800 °C, confirming the excellent thermal stability of the highly imidized structure. In air, T_d,5%_ and T_d,10%_ are 488.18 °C and 533.81 °C, respectively. Although slightly lower than those in nitrogen, these values remain significantly higher than those of conventional flame-retardant fibers such as Nomex (T_d,onset_ ≈ 370 °C), demonstrating the superior thermo-oxidative stability of the BTDA-ODA PI fibers. The maximum degradation temperatures (T_max_) are 584.41 °C in nitrogen and 636.43 °C in air, corresponding to the main chain scission of the imide rings. The complete decomposition at 800 °C in air (char residue ~0%) is characteristic of PI materials under oxidative atmospheres.

Furthermore, the flame-retardant performance was evaluated by a vertical burning test. As illustrated in Figure 7c, both PI and aramid 1313 self-extinguish within 5 s with preserved structural integrity. By contrast, polyester sustains continuous combustion accompanied by melt dripping until complete burnout. These results confirm that PI possesses intrinsic self-extinguishing properties equivalent to aramid 1313 and significantly superior to polyester, demonstrating its potential for fire-protection applications where both thermal stability and flame retardancy are critical.

The superior performance of the BTDA-ODA PI fibers originates from the synergy between chemical and structural factors. Chemically, the rigid BTDA units and flexible ODA linkages impart high thermal stability and good toughness, while nearly complete imidization (ID = 94.2%) minimizes structural defects. Structurally, pore architecture governs thermal insulation: macroporous structures with a high open area fraction interrupt solid conduction (20/80), whereas overly fine pores enhance solid conduction (50/50). The 40/60 composition achieves the optimal balance, offering competitive thermal insulation along with the highest toughness and elongation, making it the most promising candidate for thermal protection applications.

## 3. Materials and Methods

### 3.1. Materials

3,3′,4,4′-Benzophenonetetracarboxylic dianhydride (BTDA, 99% purity) was supplied by Shanghai Eien Chemical Technology Co., Ltd. (Shanghai, China). 4,4′-Diaminodiphenyl ether (ODA, 99% purity) was obtained from Jingmen Dongxin Biotechnology Co., Ltd. (Jingmen, China). N,N-Dimethylacetamide (DMAc, ≥99.8% purity) was purchased from Shanghai Aladdin Biochemical Technology Co., Ltd. (Shanghai, China). Prior to use, the solvent was rigorously dried using 4 Å molecular sieves with a soaking duration of more than 48 h. Deionized water adopted in the whole experiment was self-prepared in the laboratory. All reagents were of analytical grade.

### 3.2. Synthesis of Spinning Solutions

**Figure 8:**
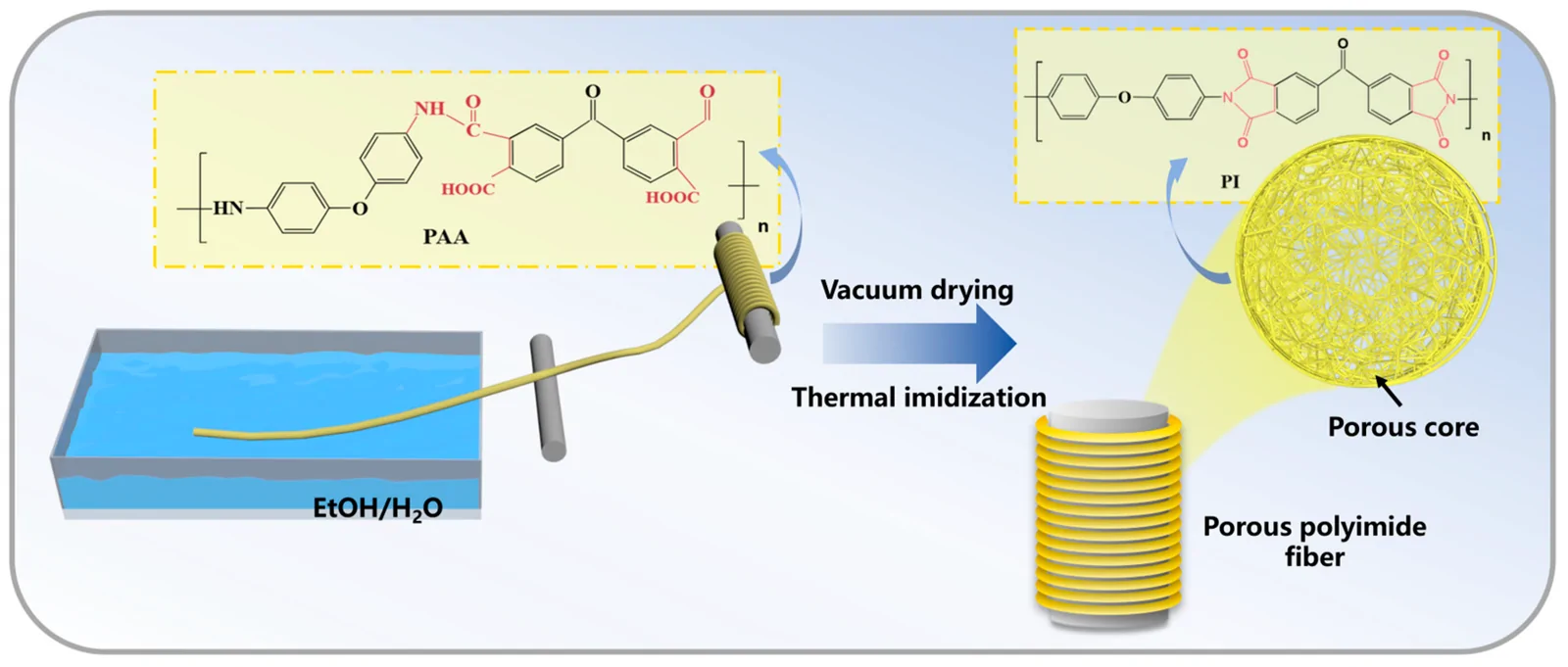
Schematic diagram of porous PI fiber fabrication.

The PAA spinning solution was synthesized via a typical polycondensation reaction, as schematically illustrated in Figure 8. Briefly, a certain amount of 4,4′-diaminodiphenyl ether (ODA) was completely dissolved in N,N dimethylacetamide (DMAc) within a three-necked round-bottom flask under continuous mechanical stirring. Subsequently, 3,3′,4,4′-benzophenonetetracarboxylic dianhydride (BTDA) powder was added in batches over 15–20 min, followed by additional DMAc to adjust the solid content of the reaction system. The mixture was continuously stirred at 0 °C for 4–5 h to complete the polymerization reaction. The molar ratio of BTDA to ODA was fixed at 1.01:1, and the final solid content of the PAA spinning solution was controlled at 15 wt%, yielding a pale yellow and viscous polymer solution.

### 3.3. Wet Spinning Preparation of PI Fibers

The homogeneous PAA solution was injected into an ethanol/water coagulation bath at an extrusion speed (V_e_) of 8 mL h^−1^ with appropriate tension applied to the fibers. A 21 G needle with an inner diameter of 0.5 mm was adopted for extrusion, and the coagulation bath temperature was maintained at 25 °C. After solidification and shaping, the as-spun fibers were collected by a winding roller with preliminary drawing, followed by immersion in a solvent exchange bath for 24 h to remove residual solvent. Subsequently, the fibers were vacuum-dried at 60 °C and further thermally imidized under nitrogen protection using a stepwise heating procedure: holding at 60 °C for 20 min, 100 °C for 100 min, and 300 °C for 30 min, with a constant heating rate of 2 °C min^−1^. The final PI fibers were successfully fabricated through the above procedures, and the overall experimental process is shown in Figure 8.

To investigate the independent effect of winding speed (V_w_) on fiber microstructure and properties, PI fibers were produced at different V_w_ of 2.6, 5.2, 7.9, 10.5, and 13.1 mm·s^−1^, while the extrusion speed was fixed at 8 mL·h^−1^ and the coagulation bath (EtOH/H_2_O) ratio at 40/60. Furthermore, to explore the influence of the coagulation bath composition, a series of EtOH/H_2_O coagulation baths with volume ratios of 20/80, 30/70, 40/60, 50/50, and 60/40 were employed under identical fixed conditions (V_e_ = 8 mL·h^−1^, V_w_ = 7.9 mm·s^−1^). All resulting fibers underwent the same post-treatment and thermal imidization procedures to ensure consistent experimental conditions.

### 3.4. Characterization

#### 3.4.1. Morphological Characterization (SEM)

Surface and cross-sectional morphologies were examined via cold-field (S-4800) and high-resolution (Regulus 8100) FE-SEM (Hitachi High-Tech Corporation, Tokyo, Japan). Samples were gold-sputtered (~5 nm) to avoid charging. Observations were performed at 5 kV accelerating voltage and 10 µA emission current.

Quantitative pore structure parameters, including pore size distribution, area porosity, and circularity, were obtained by analyzing SEM cross-sectional images using ImageJ (version 1.53e, NIH, USA). For each sample, at least three images from different regions were analyzed. The images were converted to 8-bit grayscale, and an appropriate threshold was applied to distinguish pores from the polymer matrix. The pore size distribution, area porosity, and circularity were then calculated using the Analyze Particles function.

#### 3.4.2. Mechanical Properties

Mechanical properties were tested on an Instron 3365 (Instron, Norwood, MA, USA) with a gauge length of 2 cm, a strain rate of 1 mm·min^−1^, and at least 3 replicates per group; average tensile strength and elongation at break are reported. Tests were conducted at 23 ± 2 °C and 50 ± 5% relative humidity (RH).

#### 3.4.3. Thermal Properties

Thermal conductivity was measured using a KES-F7 Thermo Labo apparatus (Kato Tech Co., Kyoto, Japan) on 5 cm × 5 cm square fabric samples in the through-thickness direction. The sample was placed between the upper and lower plates of the apparatus, and its thickness was measured under a standard compressive pressure applied by the instrument. Prior to measurement, samples were conditioned at 20 ± 2 °C and 65 ± 2% RH for at least 24 h. The conductivity λ (W/(m·K)) was derived from steady-state heat flux and the temperature gradient across the sample. Each sample was tested in triplicate, and the average values are reported. The instrument was calibrated internally following the manufacturer’s procedure before each measurement.

Bulk porosity was determined by the n-butanol absorption method. The fiber samples were dried at 60 °C for 12 h under vacuum and weighed to record the dry mass (M_0_). The samples were then immersed in n-butanol for 24 h to ensure complete penetration. After removal from n-butanol, excess liquid on the surface was gently blotted with filter paper, and the wet mass (M_1_) was recorded. The porosity P was calculated using the following equation:

(4) $$P\left(\%\right)\;=\;\frac{M_{1}/\rho_{\text{n-butanol}}}{{(M}_{1}/\rho_{\text{n-butanol}})\;+\;(M_{0}/\rho_{\mathrm{PI}})}\times 100\%$$

where $\rho_{\text{n-butanol}}$ = 0.81 g·cm^−3^ is the density of n-butanol. Each sample was tested in triplicate, and the average value is reported. 

Thermal insulation performance was assessed using a hot-plate system at set temperatures of 100, 150, and 200 °C. Square samples (5 cm × 5 cm) were placed flat on the hot-plate surface with their top side exposed to ambient air. After heating for 120 s to reach a steady state, the top-side surface temperatures were monitored using an infrared thermometer and simultaneously recorded via K-type thermocouples connected to a YET-620 L thermometer (Yowexa, Shenzhen, China); thermal images were captured using a FOTRIC thermal imager (FOTRIC, Shanghai, China). Three replicates were performed for each condition, and the average surface temperature ± SD is reported.

#### 3.4.4. Chemical Structure and Thermal Stability

The chemical structure of the PI fibers was characterized by Fourier transform infrared (FTIR) spectroscopy (IRXross, Shimadzu, Kyoto, Japan) in attenuated total reflectance (ATR) mode. Spectra were recorded over the wavenumber range of 4000–400 cm^−1^ at a resolution of 4 cm^−1^, with 32 scans accumulated per sample. The imidization index (II) was calculated as the ratio of the integrated area of the imide C–N stretching band at ~1380 cm^−1^ (S_1380_) to that of the internal reference band at ~1500 cm^−1^ (S_1500_, aromatic C=C stretching):

(5) $$\mathrm{II}\;=\;\frac{S_{1380}}{S_{1500}}$$

The relative imidization degree (ID, %) was further calculated using the following equation:

(6) $$\mathrm{ID}\;(\%)\;=\;\frac{{{(S}_{1380}/S_{1500})}_{\mathrm{Sample}}}{{\left(S_{1380}/S_{1500}\right)}_{\mathrm{Standard}}}\times 100\%$$

where the standard refers to a fully imidized reference sample.

Thermal stability was evaluated by thermogravimetric analysis (TGA) using a PE Diamond TG/DTA instrument (PerkinElmer, Waltham, MA, USA). Approximately 5 mg of each sample was heated from 30 °C to 800 °C at a heating rate of 20 °C·min^−1^ under both nitrogen and air atmospheres (a flow rate of 60 mL·min^−1^) in Al_2_O_3_ crucibles, following the ASTM E1131 standard.

## 4. Conclusions

In this work, single-component BTDA-ODA polyimide (PI) porous fibers were successfully fabricated via wet spinning. By adjusting the EtOH/H_2_O coagulation bath ratio (20/80–60/40) and winding speed (2.6–13.1 mm/s), the fiber morphology was effectively tuned from finger-like macrovoids to uniform sponge-like networks, with fiber diameters precisely controlled within 120–335 μm. The optimized PI fibers exhibited good mechanical properties, achieving a maximum tensile strength of 56.79 MPa, an elongation at break of 13.89%, and a toughness of 4.98 MJ/m^3^. The highly imidized structure was confirmed by FTIR (imidization index = 0.848, relative imidization degree = 94.2%), and TGA revealed high thermal stability with 5% weight loss temperatures of 491 °C (N_2_) and 488 °C (air). The unique porous structure imparted PI fibers with a low thermal conductivity of 0.043 W/(m·K). The fibers obtained at an EtOH/H_2_O ratio of 20/80 showed the best insulation, attributed to the high air-volume fraction from macrovoids. A comprehensive evaluation of mechanical and thermal performance identified the 40/60 ratio as the optimal coagulation bath condition, offering the best balance between toughness (4.98 MJ/m^3^) and thermal conductivity (0.050 W/(m·K)). Comparative tests with commercial materials highlight the competitive performance of the as-prepared PI fabrics under the specific conditions tested. Under hot-plate temperatures of 100–200 °C, a single-layer PI fabric (0.892 mm) achieved surface temperatures comparable to those of aramid 1313 fabric (1.588 mm), demonstrating equivalent thermal protection despite being 44% thinner. More importantly, a 5-layer PI stack (3.637 mm) was only about half the thickness of glass fiber cotton (7.342 mm) while retaining 84–91% of its temperature difference, delivering 1.7–1.8 times the thickness-normalized insulation efficiency of glass fiber cotton. These results demonstrate that the ultrathin porous PI fabric offers thermal protection comparable to that of commercial aramid and a higher thickness-normalized insulation efficiency than glass fiber cotton under the specific test conditions employed. This work provides a reliable experimental basis for designing high-performance PI fibers with dense skin and porous core structures, showing promising applications in firefighter protective clothing and electronic thermal insulation devices.

## Figures and Tables

**Table 1:** Comparison of mechanical properties of PI fibers and commercial fibers.

Material	Stress at Break (MPa)	Elongation at Break (%)	Refs.
this work	42.20–48.50	11.93–13.89	——
High-tenacity PET fiber	~1100	~10–14	[35]
Aramid 1313 (Nomex^®^, DuPont) ^a^	~400–600	~15–25	[36]
Aramid 1414 (Kevlar^®^, DuPont) ^a^	~2920	~3.6	[37]

^a^ DuPont, Wilmington, DE, USA. The data for these commercial fibers were cited from Refs. [36,37].

**Table 2:** Quantitative pore structure parameters of PI fiber obtained from SEM image analysis (ImageJ).

Coagulation Bath (EtOH/H_2_O)	Pore Diameter (nm)	Area Porosity (%)	Open Area Fraction (%)	Average Circularity
20/80	108.1 ± 155.9	47.04	34.71	0.836 ± 0.515
30/70	93.6 ± 123.7	25.55	20.34	0.819 ± 0.517
40/60	71.4 ± 125.0	28.84	24.51	0.842 ± 0.507
50/50	48.1 ± 57.4	26.52	21.14	0.868 ± 0.500
60/40	89.7 ± 142.3	26.63	23.27	0.790 ± 0.519

Note: Values were obtained by ImageJ analysis of SEM cross-sectional images and represent the 2D open area fraction on the selected cross-sections. They are used for morphological comparison across samples and should not be regarded as an absolute measure of 3D porosity.

**Table 3:** Surface temperatures (°C) of PI fiber fabrics heated at 100–200 °C under different coagulation bath conditions.

Coagulation Bath Ratio (EtOH/H_2_O)	Surface Temperature (°C)		
	100 °C	150 °C	200 °C
20/80	90.48 ± 0.83 °C	136.60 ± 2.94 °C	182.75 ± 3.46 °C
30/70	94.59 ± 1.73 °C	143.50 ± 2.04 °C	187.97 ± 3.90 °C
40/60	94.99 ± 1.59 °C	143.90 ± 1.64 °C	188.97 ± 4.49 °C
50/50	95.08 ± 1.76 °C	139.96 ± 2.56 °C	183.01 ± 2.79 °C
60/40	95.39 ± 1.25 °C	143.28 ± 1.90 °C	188.69 ± 3.50 °C

**Table 4:** Fabric thickness and thermal conductivity under different coagulation bath conditions.

Coagulation Bath Ratio (EtOH/H_2_O)	Thickness (mm)	Porosity (%)	Thermal Conductivity (W·m^−1^·K^−1^)	Thermal Resistance (m^2^·K·W^−1^)
20/80	0.847	59.55	0.043	0.020
30/70	0.834	77.05	0.044	0.019
40/60	0.820	61.93	0.050	0.016
50/50	0.748	45.33	0.053	0.014
60/40	0.894	70.45	0.052	0.017

Note: The combined relative uncertainty of the thermal conductivity measurements, arising from instrument accuracy (±3%), sample thickness variation (±0.01 mm), and temperature fluctuations (±0.5 °C), is estimated to be approximately ±5%.

**Table 5:** Theoretical bounds and effective solid thermal conductivity of PI fiber samples.

Coagulation Bath (EtOH/H_2_O)	φ (%)	λ_parallel_ (W·m^−1^·K^−1^)	λ_series_ (W·m^−1^·K^−1^)	λ_measured_ (W·m^−1^·K^−1^)	λ_eff_ (W·m^−1^·K^−1^)	λ_eff_/λ_solid_ (%)
20/80	59.55	0.117	0.041	0.043	0.068	27
30/70	77.05	0.077	0.033	0.044	0.104	42
40/60	61.93	0.111	0.04	0.05	0.089	36
50/50	45.33	0.148	0.051	0.053	0.075	30
60/40	70.45	0.092	0.035	0.052	0.114	46

Note: φ is the bulk porosity (n-butanol method, Table 4). ImageJ-derived area fractions (Table 2) are excluded because they yield physically impossible series limits (λ_series_ > λ_measured_ for all samples). λ_air_ = 0.026 W·m^−1^·K^−1^; λ_solid_ = 0.25 W·m^−1^·K^−1^. The parallel and series models are used as bounding estimates. The back-calculated λ_eff_ values are intended for relative comparison across samples rather than as an absolute determination of solid-phase thermal conductivity.

**Table 6:** Thermal degradation characteristic parameters of PI fibers in N_2_ and air atmosphere.

Parameter	N_2_	Air
T_d,5%_	491.47 °C	488.18 °C
T_d,10%_	534.02 °C	533.81 °C
T_max_	584.41 °C	636.43 °C
Char residue at 800 °C	~59.5%	~0%
Mass loss in the 100–150 °C range	<0.02%	<0.02%

## Data Availability

The original contributions presented in this study are included in the article. Further enquiries can be directed to the corresponding author.
